# Identification of a disulfidptosis-correlated ferroptosis prognostic model in breast cancer

**DOI:** 10.1097/MD.0000000000042168

**Published:** 2025-07-18

**Authors:** Tao Hu, Biao-Feng Shan, Jing-Hao Xu, Tong-Xu Zeng, Le Zhao, Bo Yu

**Affiliations:** a First Clinical Medical College, Gansu University of Chinese Medicine, Lanzhou, China; b Department of Thyroid and Breast Surgery, The Second People’s Hospital of Lanzhou City, Lanzhou, China.

**Keywords:** ANO6, breast cancer, disulfidptosis, ferroptosis, prognostic model

## Abstract

Targeting the urgent need for diagnostic biomarkers in breast cancer, the most common female malignancy, this study evaluates the **diagnostic and prognostic potential** of disulfidptosis-related ferroptosis genes (DRFGs), leveraging the emerging roles of disulfidptosis and ferroptosis in cancer biology. Ferroptosis- and disulfidptosis-related genes of patients with breast cancer were collected from The Cancer Genome Atlas database, then 154 identified prognosis-associated DRFGs were analyzed using Pearson correlation analysis, and developed a DRFG-associated risk model containing 19 DRFGs by applying the least absolute shrinkage and selection operator Cox regression analysis. We then assessed the prognostic performance of the risk model between the high- and low-risk groups. The risk scores and clinical variables were combined to construct a nomogram. Bioinformatics analyses including functional enrichment analysis and protein–protein interaction networks were conducted. Besides, we examined the biological functions, immune checkpoints, and drug sensitivity of the model. Finally, **ANO6 overexpression effects on breast cancer cell invasion and metastasis were examined via wound healing and Transwell assays.** The high-risk group showed poorer overall survival (OS) rates (*P* <.001) and lower receiver operating characteristic curve area under receiver operating characteristic values compared to the low-risk group. The results of univariate and multivariate Cox regression analyses confirmed that the established nomogram model served as an independent prognostic indicator. Functional enrichment analysis revealed the involvement of DRFGs in biological processes and signaling pathways beyond disulfidptosis and ferroptosis. The level of expressions of immune checkpoints for TDO2 and PVR were increased in the high-risk group. Drug sensitivity analysis showed that high-risk patients benefited more from AKT.inhibitor.VIII. Moreover, anoctamin 6 overexpression inhibited breast cancer cell invasion and metastasis. This study successfully established a DRFG-related prognostic model for patients with breast cancer, and anoctamin 6 has emerged as a promising therapeutic target for breast cancer.

## 1. Introduction

Breast cancer is characterized by the unregulated growth of abnormal cells in the breast, leading to tumor development. Currently, it is the fifth leading cause of death globally.^[1]^ The selection of therapy for breast cancer depends on various factors, including cancer stage, patient’s overall health, and other considerations. Although surgery, chemotherapy, radiotherapy, endocrine therapy, and targeted therapy have all shown progress in the treatment of early-stage breast cancer, the prognosis of patients with advanced breast cancer remains unclear. The diverse nature of patients contributes to the variable outcomes following combination therapy. The intricacy and genetic complexity of breast tumors may account for the constraints of current treatment choices.^[2–5]^ Therefore, there is an urgent need to formulate a novel prognostic model capable of accurately predicting breast cancer progression, with the goal of enabling personalized and effective treatments tailored to individual patients. Further exploration of the molecular mechanisms underlying breast cancer progression is imperative for the development of innovative targeted therapies.

Ferroptosis, which occurs as a result of the excessive accumulation of iron and peroxides derived from polyunsaturated fatty acids,^[6]^ is a significant factor in cancer advancement, particularly in the case of drug-resistant tumors.^[7,8]^ Studies have indicated that ferroptosis is relevant to various aspects of breast cancer, including proliferation, invasion, metastasis, and drug resistance.^[9–11]^ Furthermore, a predictive ferroptosis correlation model has been created to assess the prognosis of breast cancer cases^[12]^ as well as cases of lung squamous cell carcinoma^[13]^ and other malignancies. Disulfidptosis, a recently recognized form of cellular demise, arises from the accumulation of substantial quantities of disulfides (such as cysteine) inside the cells.^[14]^ Extracellular cysteine is actively taken up by cancer cells and subsequently converted to cystine via an intracellular NADph-dependent mechanism.^[15]^ The abnormal buildup of disulfides (e.g., cystine) within cells can trigger severe disulfide stress, resulting in high cytotoxicity.^[16]^ Excessive cystine accumulation within cells is linked to cellular demise.^[17]^ The antitumor properties of cystine have been observed in diverse cancers, suggesting potential applications in cancer therapy.^[18]^ Disulfidptosis has been shown to inhibit the growth of breast cancer cells and thereby influence cancer progression.^[19]^ The results of previously published studies have indicated that the disulfidptosis correlation model is a reliable predictor of the prognosis of liver cancer,^[20]^ pancreatic cancer,^[21]^ breast cancer,^[19,20]^ and other malignancies. Recent investigations revealed that a combination of these 2 cell death mechanisms enhances the accuracy of cancer prognosis.^[22,23]^ Moreover, the incorporation of multiple cell death modes in breast cancer has resulted in superior prognostic efficacy.^[24,25]^ Consequently, it is plausible to anticipate that merging ferroptosis and disulfidptosis could offer novel insights into the diagnosis and treatment of this disease.

Anoctamin 6 (ANO6, also known as TMEM16F) is a transmembrane protein that participates in ferroptosis and disulfidptosis. ANO6 is implicated in the rearrangement of phospholipids and facilitation of cell migration. In pancreatic cancer, ANO6 stimulates growth and suppresses immune response.^[26]^ In gliomas, ANO6 enhances cell proliferation and invasion by modulating the ERK signaling pathway.^[27]^ However, the precise mechanism underlying the action of ANO6 in BC remains unclear.

The findings in the present study are highly beneficial in predicting disease outcomes in patients with breast cancer. Furthermore, we discovered a connection between the immune atmosphere and the breast cancer prognostic model, which supports the justification of therapeutic interventions targeting the immune system. We also identified a pharmaceutical compound that manifested heightened responsiveness in high-risk cohorts. Through experimental validation, we found that excessive expression of ANO6 profoundly impedes the migration of breast cancer cells. These findings substantiate the involvement of disulfidptosis-related ferroptosis genes (DRFGs) in breast cancer and serve as the basis of a new framework for tailoring precise treatments for patients with breast cancer.

## 2. Materials and methods

### 2.1. Data extraction and screening of differentially expressed DRFGs

We collected from The Cancer Genome Atlas (TCGA; https://portal.gdc.cancer.gov) the gene expression profiles and clinicopathological parameters of 113 breast tissues considered to be in a normal state and 1109 tissues affected by breast cancer. To explore the relationship between disulfidptosis and gene expression, we compiled a list of 23 genes associated with disulfidptosis based on a previous study.^[14]^ We obtained a comprehensive set of 564 ferroptosis-related genes from the FerrDb V2 database (http://www.zhounan.org/ferrdb/).^[28]^ To identify DRFGs, Pearson correlation analysis was performed, considering only those genes with a correlation coefficient |*R*^2^| >.2 and *P* < .05. Then we filtered out differentially expressed DRFGs between normal and BC tissues in |log₂FC| >.5 and *P* < .05.

### 2.2. Functional enrichment analysis and PPI network construction

To conduct the analysis of gene ontology (GO) and Kyoto Encyclopedia of Genes and Genomes (KEGG), the “clusterProfiler,” “enrichplot,” and “ggplot2” packages were utilized within the R programming language. Furthermore, interaction networks involving proteins were obtained from the STRING database (https://cn.string-db.org/) and visualized using the Cytoscape software. To identify the most significant hub genes, the maximal clique centrality method was applied using the Cytohubba plugin, resulting in the selection of the top 10 hub genes.

### 2.3. Establishing and validating the prognostic model of DRFGs

Univariate Cox regression analysis was used to identify the DRFGs associated with prognosis. The “glmnet” package was employed in the construction of a risk model through least absolute shrinkage and selection operator (LASSO) Cox regression analysis. The risk score was calculated as follows: risk score = (Coef 1 × expression mRNA1) + (Coef 2 × expression mRNA2) + (Coef n × expression mRNA n). Based on the intermediate-risk scores, patients with breast cancer were categorized into high- and low-risk groups. The Kaplan–Meier curve was used for survival analysis. Receiver operating characteristic analysis was performed to evaluate the prognostic potential of the risk model. Furthermore, we validated the model’s efficacy by randomly selecting 70% of the data from the TCGA dataset.

### 2.4. Establishing a nomogram

We investigated the association between the clinical variables and the model. Cox regression analysis was performed to assess the individual prognostic value of the model, using multivariate and univariate analyses. An assessment of the probability of 1, 3, and 5-year OS of patients with breast cancer was carried out by constructing a nomogram using the “RMS” package. Calibration plots were used to validate the predictive value of the nomograms.

### 2.5. Analysis of immunity indicators and GSEA

We analyzed the expression of immune checkpoints (TDO2, PVR, PDCD1, HLAE, LGALS9, TNFRSF4, ADORA2A, and CD27) to predict the efficacy of immune-related checkpoint therapy in the model.^[29]^ Gene set enrichment analysis (GSEA) was also performed to explore the underlying physiological mechanisms of the model.

### 2.6. Drugs sensitivity analysis

To assess the semi-maximum inhibitory concentration (IC50) and predict drug sensitivity, we utilized the genomics of drug sensitivity in cancer datasets. Our analysis employed the “Prophesy” model and R programming language with the “prophetic” package to calculate the sensitivity score.

### 2.7. Cell culture and transient transfection

MDA-MB-231 cells were obtained from the cell repository of the Kunming branch of the Chinese Academy of Sciences (China). These cells were cultured in nutritive medium containing 10% fetal bovine serum (GIBCO-10099141, Australia) and 90% Dulbecco’s modified Eagle’s medium/nutrient mixture F-12 (DMEM/F-12, GIBCO-8122583, China). We used the pCDNA3.1-ANO6-HIS plasmid developed by Musculus Biotechnology (GENEWIZ, China). MDA-MB-231 cells were transfected with the plasmid pCDNA3.1-ANO6-HIS and the inert control vector pcDNA3.1 using jetPRIME transfection reagent. For subsequent functional assays, cells were transfected with various plasmid doses for 48 hours. We performed conventional western blot analysis using total cell protein extracts. For detection, we used antibodies against ANO6 (Thermo Fisher Scientific; Waltham), actin (Proteintech; Wuhan, China), and secondary Polyclonal Rabbit IgG (Kang Wei; Jiangsu, China).

### 2.8. Cell invasion and metastasis

To evaluate the invasion and metastasis capabilities of MDA-MB-231 cells with modified ANO6 expression, we conducted cell scratch and Transwell chamber experiments. In the scratch assay, 6-well plates were used to cultivate the cells until full coverage was achieved. Using a 200-μL pipette tip, lesions were induced on the surface of the cellular monolayer. Subsequently, we captured images of the compromised monolayer at 0 hour and at 24-hour intervals, and the measurement of migration was based on the gap at multiple locations. For the Transwell assay, we diluted the cellular suspension to a concentration of 5 × 10^4^/mL. Subsequently, 200 μL of this suspension was added to the apical chamber along with serum-free medium, and 600 μL of medium enriched with 10% fetal bovine serum was added to the basal chamber. After 24 hours, cells within the chamber were gently removed using a cotton swab. Cells that adhered to the lower side of the filter were fixed with 3.7% paraformaldehyde for 30 minutes and then stained with 0.1% crystal violet for 10 to 15 minutes. Following air-drying, an optical microscope was used to count the cell populations in 3 randomly selected fields.

### 2.9. Statistical analysis

The R software (versions 4.2.3 and 4.1.1; R Foundation for Statistical Computing, Vienna, Austria) was used for all statistical analyses conducted in this study. Pearson’s analysis was used to extract DRFGs, and the Chi-square test was used to explore discrepancies in clinical characteristics between patients in distinct risk groups. Univariate analysis, multivariate analysis, and LASSO shrinkage and selection operator regression were used to construct a prognostic DRFG model. Spearman’s rank correlation analysis was used to examine the correlation between the DRFG score and degree of immune infiltration. Statistical significance was set at *P* < .05.

## 3. Results

The workflow diagram for this study is shown in Figure S1, Supplemental Digital Content, https://links.lww.com/MD/O705.

### 3.1. Establishment and verification of the DRFGs-related model

**Figure 1.**
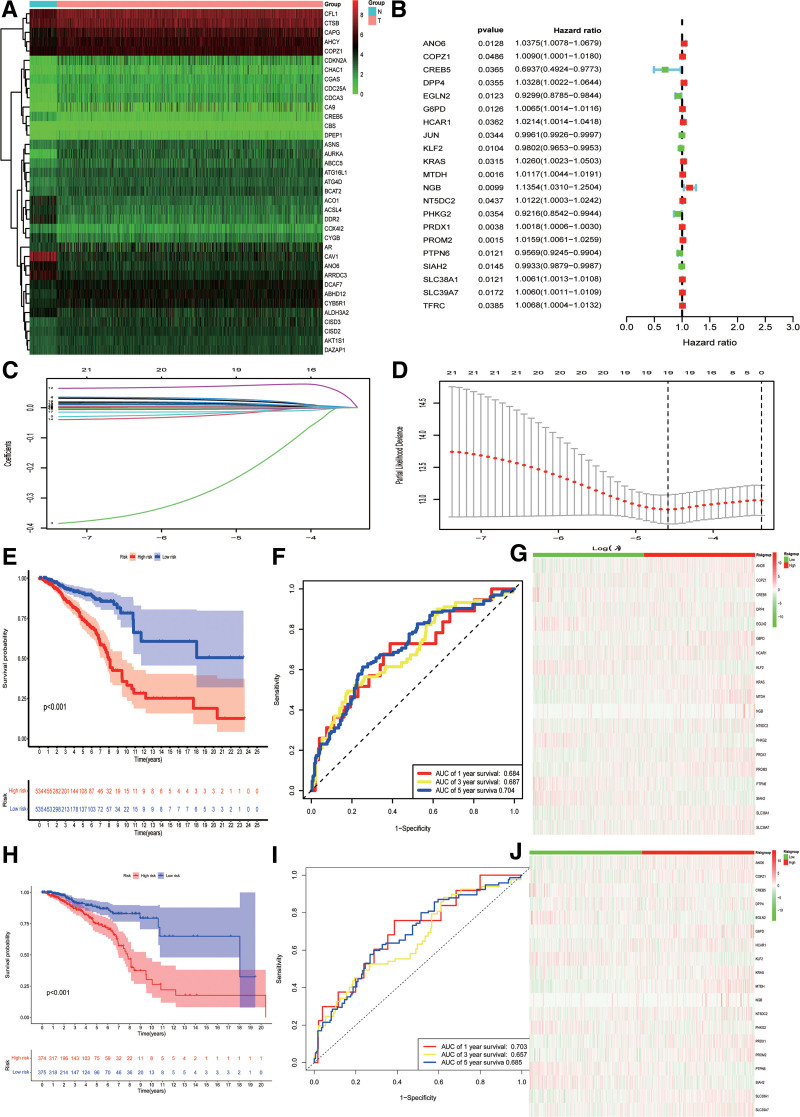
Screen prognosis-related DRFGs and constructed prognosis model. (A) Heatmap of differentially expressed DRFGs. (B) Cox regression analysis of univariate Cox regression analysis predicted the prognosis of DRFGs. (C) LASSO coefficient spectrum of 19 DRFGs. (D) Cross-validation of a proportional hazards model’s adjustment parameter choice. (E) KM analysis in the high- and low-risk groups. (F) ROC analysis for predictive capability. (G) Heatmaps of the expression difference of 19 DRFGs. (H–J) Corresponding to verification in 70% of the data in the TCGA dataset. AUC = area under ROC, DRFGs = disulfidptosis-related ferroptosis genes, KM = Kaplan–Meier, LASSO = least absolute shrinkage and selection operator, ROC = receiver operating characteristic, TCGA = The Cancer Genome Atlas.

We obtained gene expression profiles and clinicopathological parameters of 1109 breast cancer and 113 normal tissue samples (Tables S1 and S2, Supplemental Digital Content, https://links.lww.com/MD/P386; https://links.lww.com/MD/P387). A total of 23 genes associated with disulfidptosis (SLC7A11, GYS1, NDUFS1, NDUFA11, NUBPL, NCKAP1, LRPPRC, SLC3A2, RPN1, ACTN4, ACTB, CD2AP, CAPZB, DSTN, FLNA, FLNB, INF2, IQGAP1, MYH10, MYL6, MYH9, PDLIM1, TLN1) and 564 ferroptosis genes (Table S3, Supplemental Digital Content, https://links.lww.com/MD/P387) were examined. Using Pearson’s correlation analysis, we identified 353 DRFGs. Using the expression data from the breast cancer and normal breast tissues, we successfully identified 154 differentially expressed DRFGs (56 downregulated and 98 upregulated; Fig. 1A). Subsequently, we conducted univariate Cox regression analysis and identified 21 prognostically significant DRFGs (Fig. 1B). To construct a risk model, we performed a LASSO regression analysis based on the outcomes of the univariate Cox regression analysis (Fig. 1C, D). Consequently, we successfully developed a risk model comprising of 19 DRFGs. Patients with breast cancer were classified into high- and low-risk groups based on intermediate-risk scores.

An assessment was conducted to evaluate the reliability of the risk model and ascertain the accuracy of the model outputs. The group classified as high-risk exhibited lower rates of mortality and OS than did the low-risk group, indicating poorer disease outcome (*P* < .001; Fig. 1E). Furthermore, the receiver operating characteristic curves showed the model’s ability to accurately predict survival rates at 1, 3, and 5 years with levels of 0.684, 0.687, and 0.704, respectively (Fig. 1F). These findings strongly indicate the stability of the prognostic model. Additionally, heatmap analysis revealed overexpression of all genes except CAMP responsive element binding protein 5, Egl-9 family hypoxia inducible factor 2, KLF transcription factor 2, phosphorylase kinase catalytic subunit gamma 2, protein tyrosine phosphatase non-receptor type 6, and SIAH2 in the high-risk cohort (Fig. 1G). For validation purposes, we extracted 70% of the data from the TCGA dataset and conducted tests using the “caret” package. The test results corroborated those obtained from the TCGA dataset (Fig. 1H–J). Hence, the DRFGs model has immense potential for accurately predicting the prognosis of breast cancer cases.

### 3.2. Relationship of the prognostic model with clinical features

**Figure 2.**
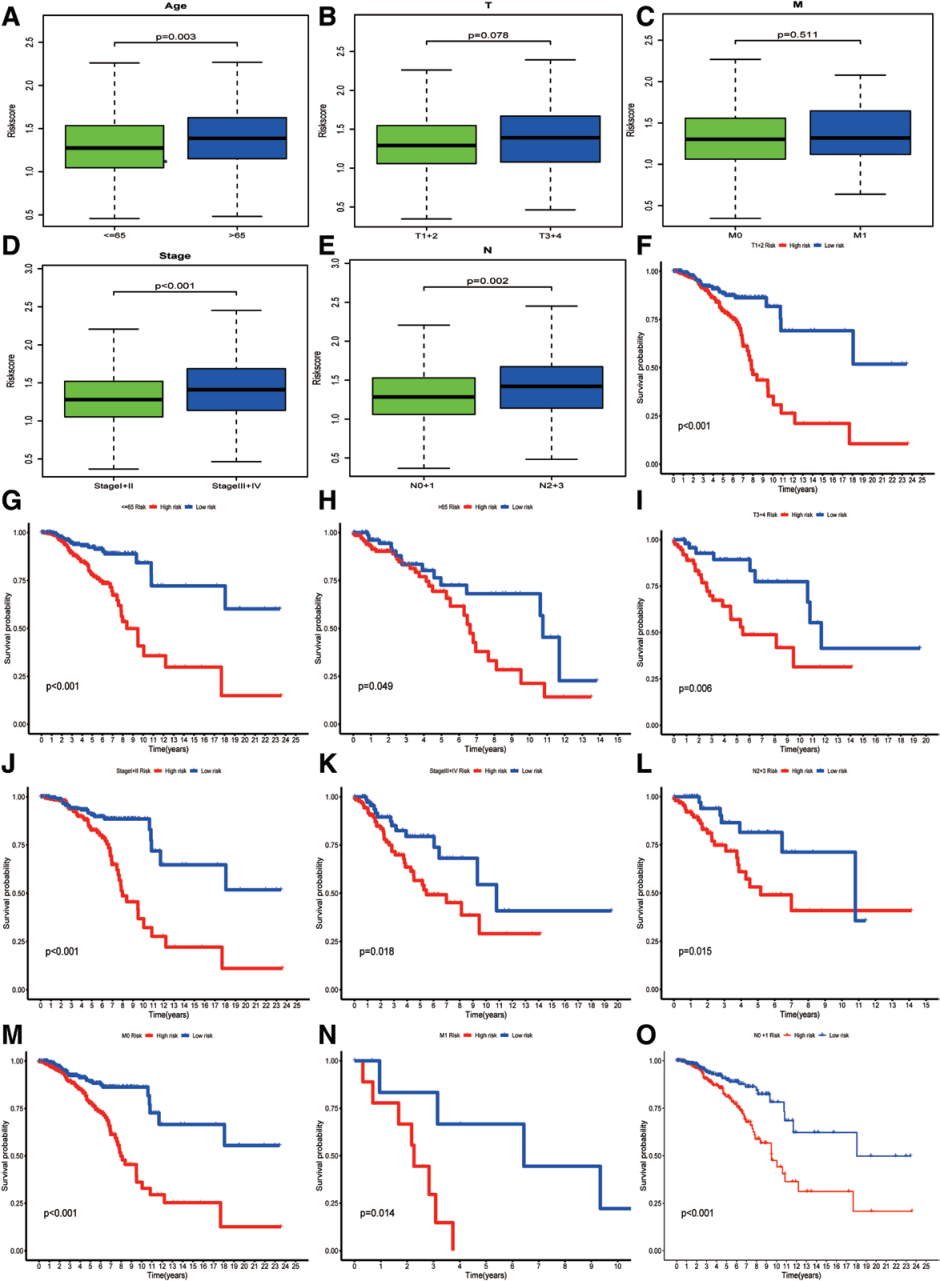
Relationship of the prognostic model with clinical features. (A–E) Correlation between risk score and clinical characteristics. (F–O) Prediction between the model and clinical features.

An investigation was conducted to examine the correlations between different clinicopathological factors and the model. The findings revealed that the high-risk group was characterized by higher values for age, stage, and lymphoid metastasis (N; *P* < .05; Fig. 2A–E). Further analyses were performed to evaluate the prognostic importance of the subgroup characteristics. The model displayed excellent predictive capability in individuals aged 65 years or older, those with M0 or M1 status, T1+2 or T3+4 tumors, N2+3 lymphoid metastasis, and stage I+II or stage III+IV (*P* < .05; Fig. 2F–O).

### 3.3. Constructing and evaluating the nomogram

**Figure 3.**
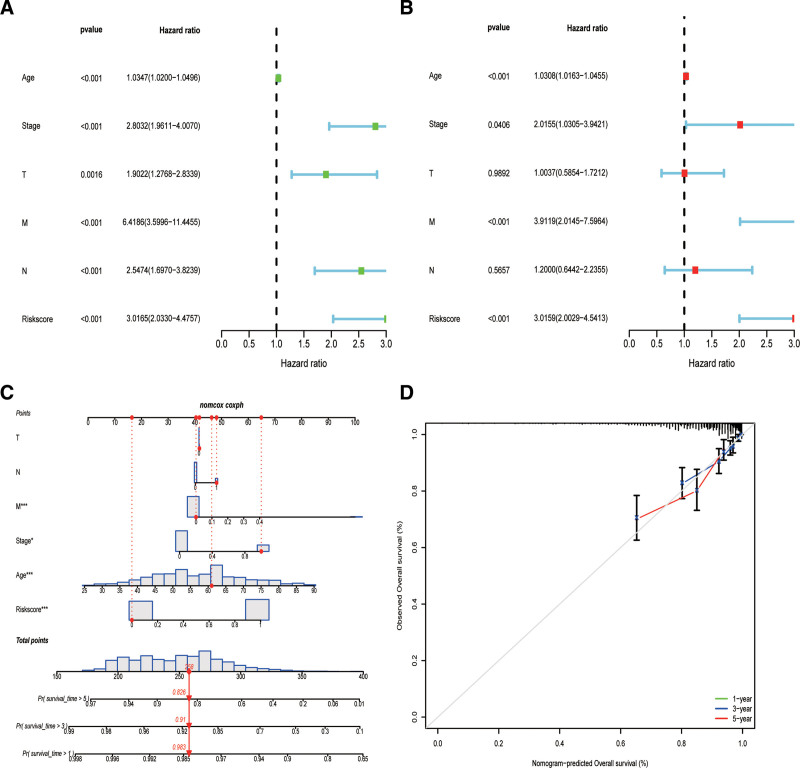
Cox regression analysis and construction of a nomogram. (A, B) Univariate and multivariate Cox regression analysis of the correlation in risk score and clinicopathological factors. (C) A nomogram for predicting 1-3-5 years of OS. (D) Calibration curve of the nomogram. OS = overall survival.

Using Cox regression analysis, we conducted a comprehensive examination of both univariate and multivariate factors. These outcomes strongly validate the reliability of our model as a prognostic indicator (Fig. 3A, B). To ensure accurate prognosis of operating systems in patients with breast cancer, we developed a column chart that integrates age, stage, N, M, T, and risk score. By referring to this chart, 1 can visually assess the survival probability of each individual by considering a variety of prognostic factors. Moreover, the constructed nomogram facilitated the prediction of survival rates at 1, 3, and 5 years in patients with breast cancer (Fig. 3C). Calibration curve analysis demonstrated a remarkable consistency between the observed and predicted survival values (Fig. 3D), further confirming the predictive capability of the nomogram. The calibration curve shows a high level of concordance between the observed and predicted values. Based on these findings, we assert that our model functions as a reliable prognostic instrument.

### 3.4. Functional enrichment analysis and PPI networks

**Figure 4.**
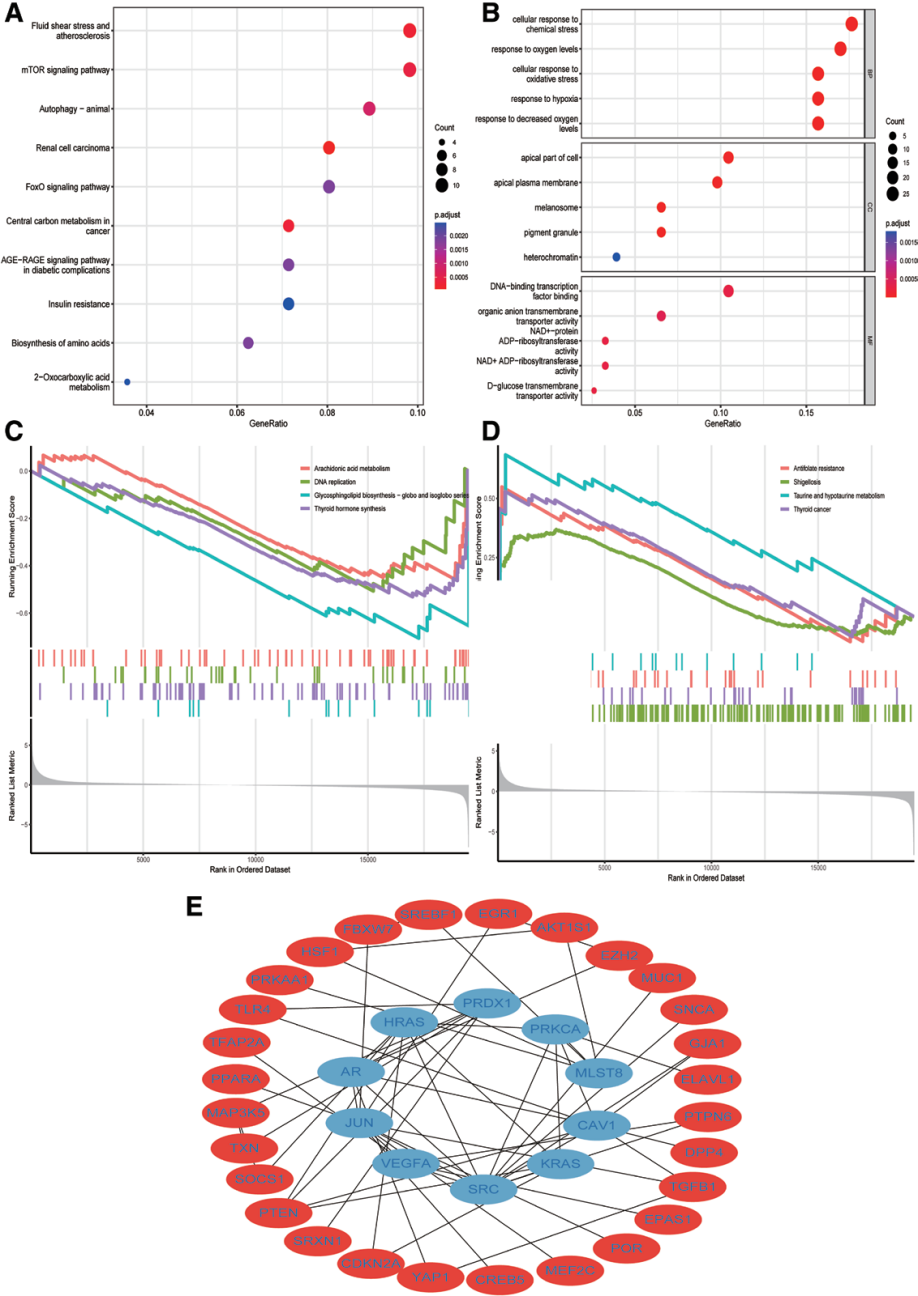
Function enrichment analysis. (A) GO. (B) KEGG. (C, D) GSEA in low and high-risk groups. (E) PPI. GO = gene ontology, GSEA = gene set enrichment analysis, KEGG = Kyoto Encyclopedia of Genes and Genomes, PPI = protein–protein interaction.

Through GO and KEGG analyses, a more comprehensive understanding of the potential roles of differentially expressed DRFGs was obtained. The analysis revealed that these DRFGs play a significant role in various biological processes, such as responding to chemical stress, changing oxygen levels, oxidative stress, hypoxia, and decreased oxygen levels. Cell component analysis demonstrated that these DRFGs were primarily located in the apical plasma membrane, melanosomes, pigment granules, apical part of the cell, and heterochromatin. In terms of molecular function, the results of our analysis indicated that these DRFGs were associated with DNA-binding transcription factor binding, organic anion transmembrane transporter activity, NAD+-protein ADP-ribosyltransferase activity, NAD+ ADP-ribosyltransferase activity, and d-glucose transmembrane transporter activity (Fig. 4A). Furthermore, KEGG analysis revealed that these DRFGs were mostly related to the mammalian target of rapamycin (mTOR), FOXO, and AGE-RAGE signaling pathways in diabetic complications, animal autophagy, fluid shear stress, atherosclerosis, and renal cell carcinoma (Fig. 4B). These findings suggest that differentially expressed DRFGs play a role beyond disulfidptosis and ferroptosis in various biological processes. In addition, GSEA was performed using this model. The results illustrated that arachidonic acid metabolism, DNA replication, glycosphingolipid biosynthesis (globo and isoglobo series), and thyroid hormone synthesis were primarily enriched in the low-risk group (Fig. 4C). Conversely, the high-risk group was enriched for antifolate resistance, shigellosis, taurine and hypotaurine metabolism, and thyroid cancer (Fig. 4D). Lastly, a PPI network was constructed based on the differentially expressed DRFGs, consisting of 154 nodes, 93 edges, and 50 expected edges (Fig. 4E).

### 3.5. Immune checkpoints

**Figure 5.**
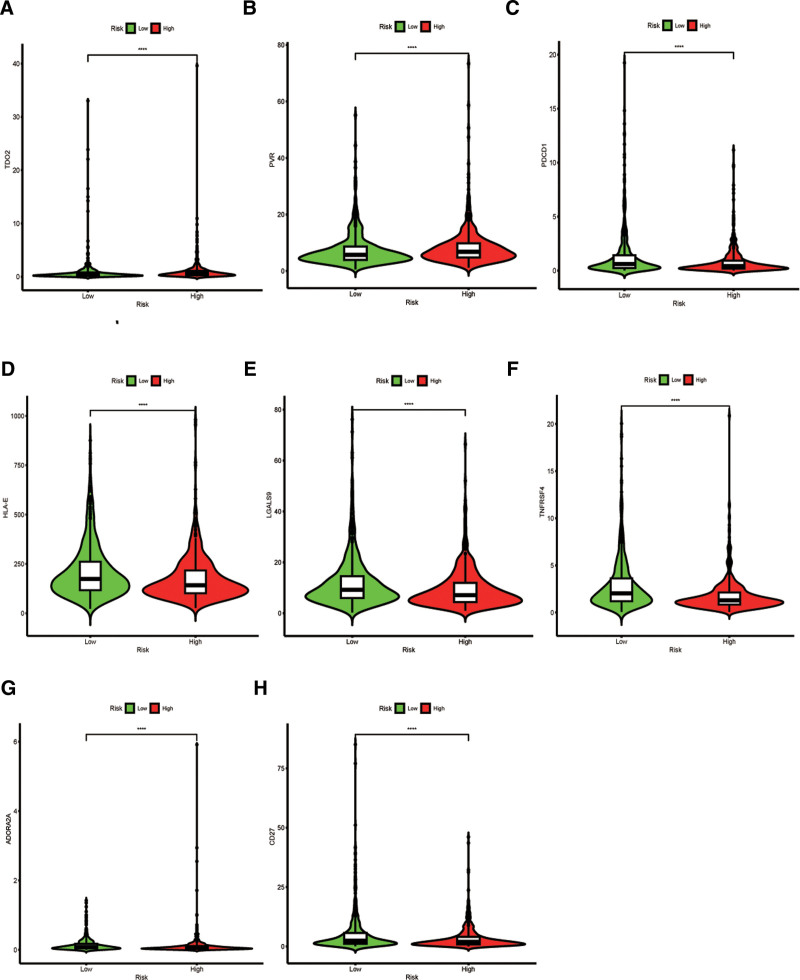
Immune checkpoints. Immune checkpoint points: (A) TDO2. (B) PVR. (C) PDCD1. (D) HLA-E. (E) LGALS9. (F) TNFRSF4. (G) ADORA2A. (H) CD27. ADORA2A = adenosine receptor subtype A2a CD27 = TNFRSF7, HLA-E = human leukocyte antigen, LGALS9 = galectin-9, PDCD1 = programmed cell death 1, PVR = CD155, TDO2 = tryptophan 2,3-dioxygenase, TNFRSF4 = tumor necrosis factor receptor superfamily, member 4.

 The correlation between the model and the immune checkpoints was examined to evaluate the effects of immunotherapy. In the high-risk group, increased expression of TDO2 and PVR was detected, implying immunosuppression (Figure 5A, B); in contrast, the low-risk group exhibited elevated expression of TNFRSF14, PDCD1, LGALS9, HLA-E, CD27, and ADORA2A (Figure 5C-H).

### 3.6. Drug sensitivity analysis

**Figure 6.**
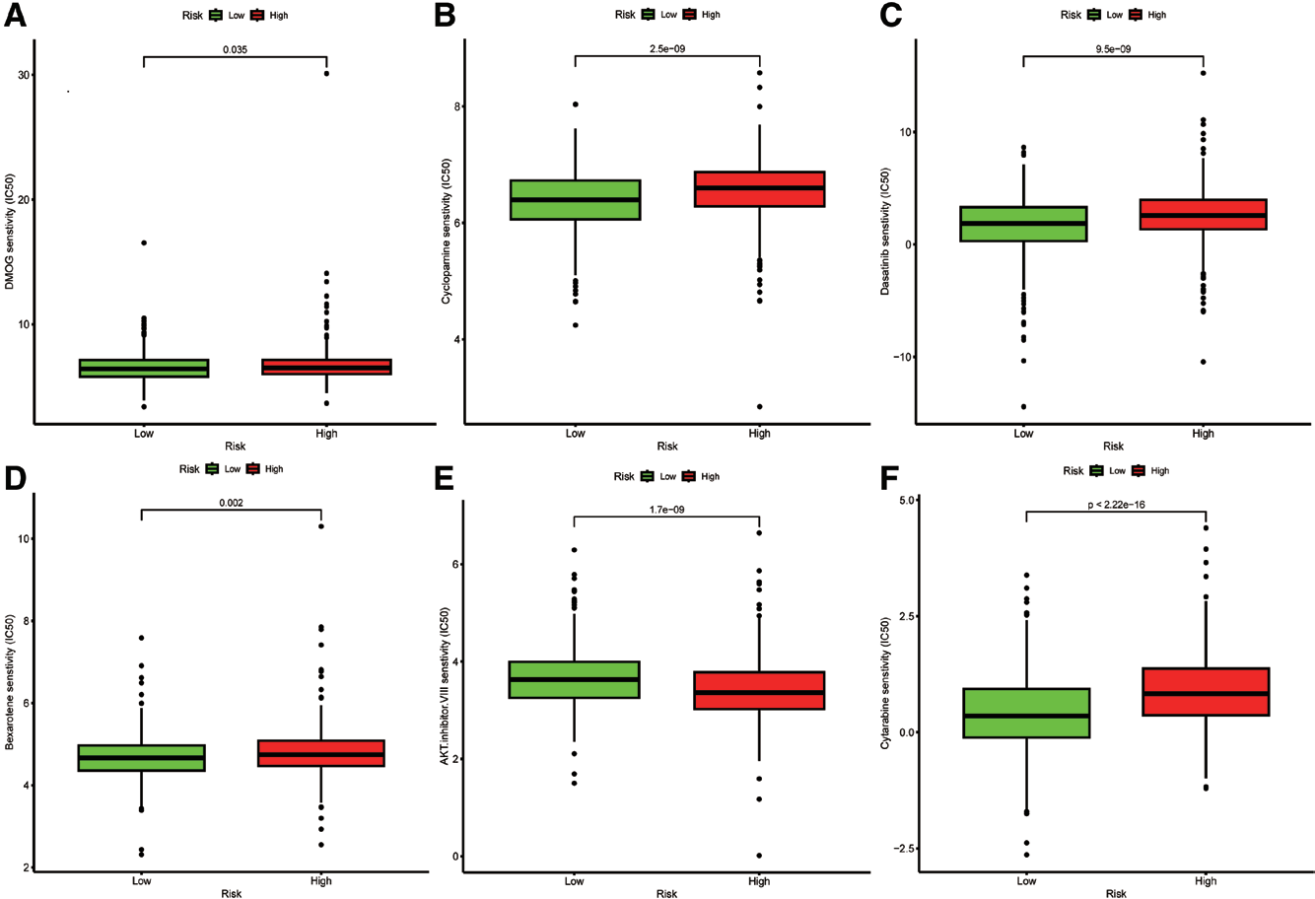
Drug sensitivity analysis. Drug sensitivity of (A) DMOG, (B) cyclopamine, (C) dasatini, (D) bexaroten, (E) AKT.inhibitor.VIII, and (F) cytarabine between the high- and low-risk groups. AKT = protein kinase B, DMOG = dimethyloxallyl glycine, IC50 = half-maximal inhibitory concentration.

Utilizing the prophetic algorithm, we employed the genomics of cancer drug sensitivity data to estimate variations in sensitivity towards frequently used chemotherapeutic agents in our model based on half-maximal inhibitory concentrations (IC50). The ultimate goal is to improve treatment outcomes in patients with breast cancer. Our findings revealed that the low-risk group demonstrated greater responsiveness to cyclopamine, cytarabine, asatinib, bexarotene, and dimethyloxallyl glycine (excluding AKT.inhibitor.VIII), as indicated by their decreased IC50 values, compared to the corresponding values in the high-risk group (Fig. 6A–F).

### 3.7. ANO6 overexpression inhibits breast cancer cell invasion and metastasis

**Figure 7.**
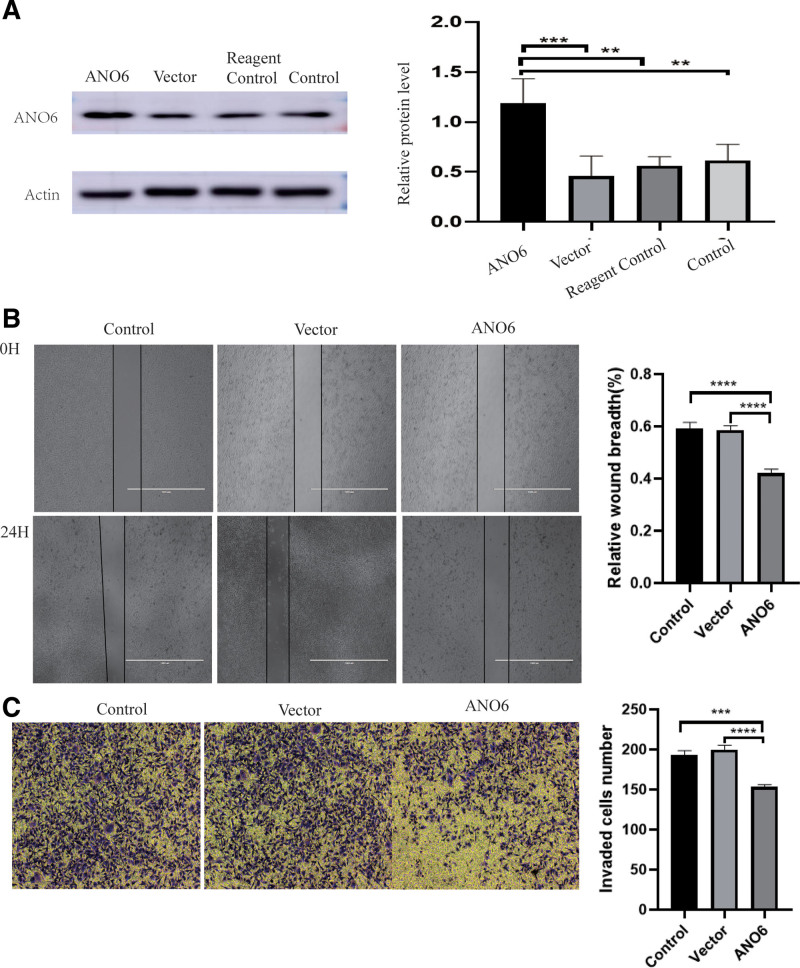
ANO6 overexpression inhibits breast cancer cell invasion and metastasis. (A) The protein expression level of ANO6 in the NC, ANO6-vector, reagent control and ANO6-overexpression. (B) Cell scratch healing tests were performed to compare the migration distance between NC, ANO6-vector, and ANO6-overexpression groups. (C) Transwell assays were performed to compare the number of cell migrations between NC, ANO6-vector, and ANO6-overexpression groups. ANO6 = anoctamin 6, ANO6-overexpression = ANO6-overexpression group, ANO6-vector = overexpression control group, NC = blank control group.

The results of the analysis reported here highlight the significant association between ANO6 and breast cancer risk as well as its strong correlation with disease outcomes. Therefore, ANO6 can be regarded as a reliable prognostic biomarker for this particular malignancy. Previous investigations have indicated that breast cancer exhibits ANO6 under-expression, suggesting its crucial involvement in disease development.^[30]^ To examine the impact of altered ANO6 expression on breast cancer biology, a series of experiments were conducted wherein ANO6 was overexpressed in comparison with the vector, reagent control, and control. To confirm this overexpression, western blot analysis revealed a substantial increase in the intracellular levels of ANO6 (Fig. 7A). Wound healing assays were performed to assess metastasis velocity, and the results demonstrated that ANO6 overexpression significantly reduced the migratory velocity of MDA-MB-231 cells compared to the effects of the vector and control (Fig. 7B). Additionally, Transwell assays provided further evidence of the hindrance of cellular invasion due to ANO6 overexpression (Fig. 7C). These findings strongly suggest that upregulation of ANO6 can impede the invasion and metastasis of breast cancer cells, thus influencing the progression of this disease.

## 4. Discussion

Searching the TCGA dataset, we successfully identified 154 differentially expressed DRFGs that exhibited differential expression between normal and breast cancer tissues. It is crucial to acknowledge the limited nature of the data employed in this study, which were derived from a preexisting public database, namely, TCGA. To better comprehend the potential functions of these DRFGs, we carried out a meticulous analysis involving KEGG and GO. Our findings suggested a strong association between these DRFGs and vital pathways, including the mTOR signaling pathway, heterochromatin regulation, and Forkhead Box O (FOXO) signaling pathways. Notably, it has been well-documented in previous studies that the mTOR signaling pathway plays a pivotal role in the initiation and progression of breast cancer.^[31]^ Moreover, FOXO dysregulation has been identified as a key factor in understanding the mechanisms underlying endocrine resistance.^[32]^ As well, deletion of heterochromatic X chromosomes has been implicated in the generation of epigenetic and genetic instability in breast cancer.^[33]^ Collectively, these investigations provide compelling evidence supporting a close association between differentially expressed DRFGs and the occurrence and progression of breast cancer.

By analyzing 1 variable, we identified and filtered out 19 functional genes that were differentially regulated and had a direct impact on the prognosis of breast cancer. Subsequently, a prognostic model for BC was created using LASSO analysis incorporating these regulated genes. Analysis using the Kaplan–Meier method showed that the high-risk group had a significantly lower survival rate. Furthermore, an examination of the relationship between the clinicopathological characteristics and the model revealed that the high-risk group had higher age, stage, and N levels, ultimately leading to lower survival rates. These results serve as strong evidence that risk score plays a significant role in breast cancer progression. Additionally, both univariate and multivariate Cox regression analyses confirmed that the developed model served as an independent prognostic indicator of disease outcome. GSEA revealed that the model was primarily associated with cancer-related metabolic pathways. Notable examples include arachidonic acid metabolism, globo- and glycosphingolipid biosynthesis, thyroid hormone synthesis, antifolate resistance, shigellosis, taurine, and hypotaurine metabolism. These findings align with those of previous studies that demonstrated the inhibitory effect of ACSL4 on arachidonic acid metabolism and tumor development.^[34]^ A connection between shigellosis and cancer progression has been reported.^[35]^ In addition, studies have revealed that thyroid hormone synthesis promotes breast cancer invasion.^[36]^ Considering these factors, we can confidently assert that our model has great potential to enhance our understanding of breast cancer biology.

Evaluating the immune system of the human body can facilitate the development of individualized treatment strategies for patients with breast cancer.^[37]^ .We explored the correlation between the model and crucial immune checkpoints. We observed upregulation of TDO2 and PVR in the high-risk group, suggesting immunosuppression. Previous studies have demonstrated the ability of TDO2 inhibitors to inhibit lung metastasis in BC.^[38]^ Moreover, we identified several potential therapies for the low-risk group including cyclopamine, cytarabine, dasatinib, bexarotene, and dimethyloxallyl glycine. Conversely, AKT inhibitors (VIII) may be advantageous in high-risk patients. It has been demonstrated that AKT inhibitors (VIII) can impede PKB phosphorylation in BC patients with HER2(ErbB2)-positive tumors.^[39]^

ANO6 is a protein found in the cell membrane that has been linked to various forms of cancer, such as glioma,^[27]^ pancreatic ductal adenocarcinoma,^[40]^ gastric adenocarcinoma,^[41]^ lung adenocarcinoma,^[42]^ and neuroblastoma.^[43]^ Despite previous studies indicating that ANO6 is less active in breast cancer, a comprehensive understanding of its specific mechanism of action in this malignancy is still lacking. Our experiments showed that increased ANO6 expression impedes the invasion and metastasis of breast cancer cells, thus significantly influencing disease progression.

There were some limitations in this study. First, the use of an established database may have affected the reliability of the outcomes. It is imperative to conduct additional investigations involving individual cohorts and experiments with the DRFGs function for predictive forecasting to affirm the validity of our findings. As well, the statistical significance of our findings was constrained by inadequate data availability and a limited sample size. Last, further in-depth studies are still necessary to investigate the underlying mechanism concerning the regulatory role of ANO6 in ferroptosis in BC.

## 5. Conclusions

A prognostic model based on DRFGs was devised to predict disease outcomes in patients with breast cancer. This model functions as an individual prognostic determinant and exhibits a robust correlation with clinical traits. Directing attention to DRFGs may offer a promising approach to combating BC. The hindrance of breast cancer invasion and metastasis – and, consequently, a slowing of disease progression and improved outcomes for the patient – may be possible with treatments based on overexpression of ANO6.

## Author contributions

**Data curation:** Jing-Hao Xu, Tong-Xu Zeng.

**Methodology:** Tao Hu, Biao-Feng Shan, Bo Yu.

**Project administration:** Bo Yu.

**Software:** Tao Hu, Biao-Feng Shan.

**Validation:** Tao Hu.

**Writing – original draft:** Tao Hu, Biao-Feng Shan.

**Writing – review & editing:** Jing-Hao Xu, Tong-Xu Zeng, Le Zhao.

## Supplementary Material


