# Supplementary figures and images for: Identification of a disulfidptosis-correlated ferroptosis prognostic model in breast cancer
Source: Medicine (Baltimore). 2025 Jul 18;104(29):e42168. doi: 10.1097/MD.0000000000042168 (PMC12282832; doi:10.1097/MD.0000000000042168)

Figure ​S1: The workflow diagram.


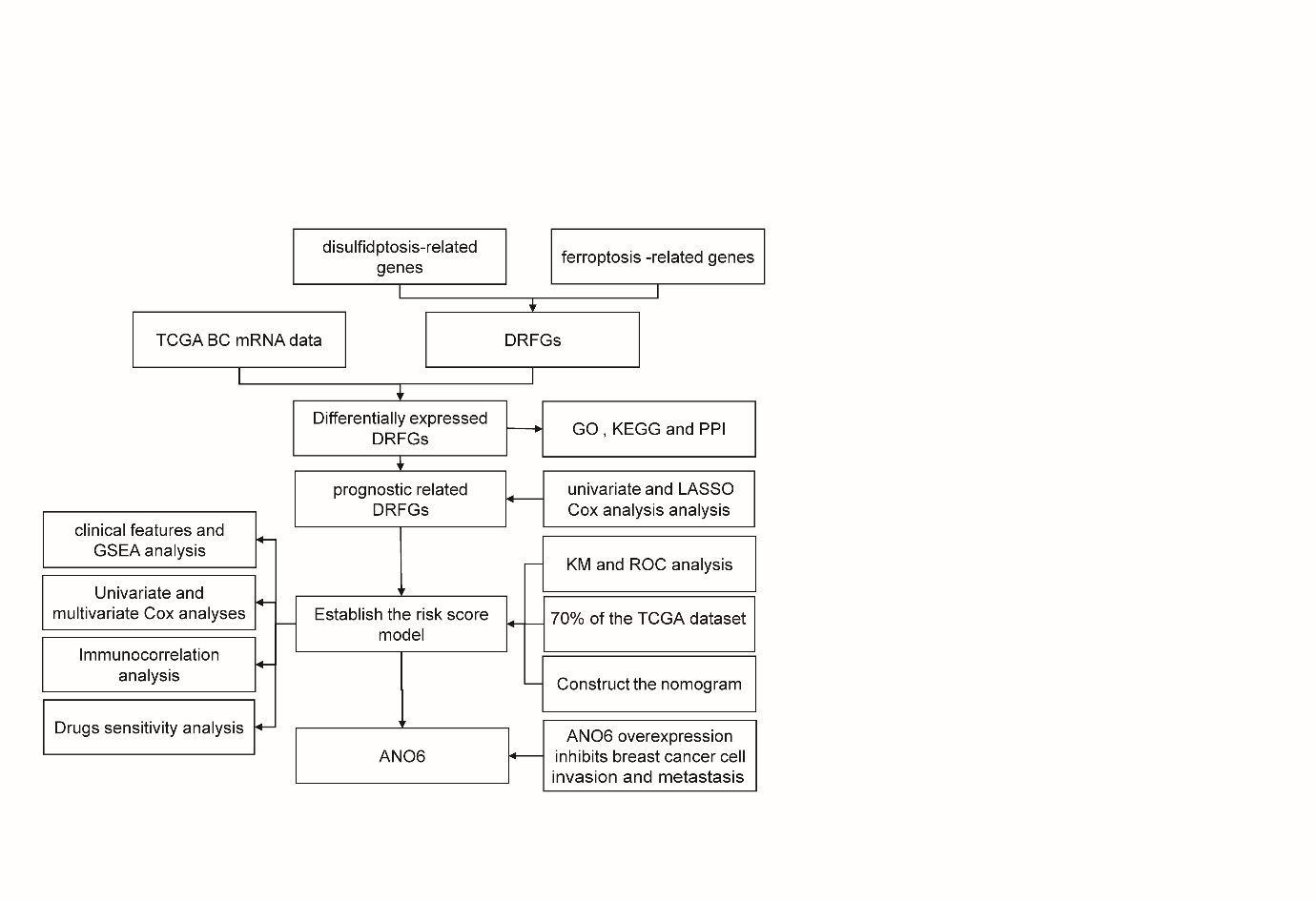

Supplement: Supplementary file 1 [file medi-104-e42168-s001.docx]
