# Supplementary material for: Identification of a disulfidptosis-correlated ferroptosis prognostic model in breast cancer
Source: Medicine (Baltimore). 2025 Jul 18;104(29):e42168. doi: 10.1097/MD.0000000000042168 (PMC12282832; doi:10.1097/MD.0000000000042168)
Supplement: Supplementary file 2 [file medi-104-e42168-s002.docx]

Table S1: BC gene expression profile from TCGA.

| gene | group1mea | group2mea | logFC | p | FDR |
| --- | --- | --- | --- | --- | --- |
| ABCC1 | 7.513931 | 9.694996 | 0.367672 | 1.81E-08 | 3.09E-08 |
| ABCC5 | 3.522616 | 7.686751 | 1.125726 | 6.76E-17 | 1.69E-16 |
| ABHD12 | 12.61615 | 27.17417 | 1.106964 | 3.01E-48 | 4.43E-47 |
| ACADSB | 18.82255 | 19.05101 | 0.017405 | 0.002839 | 0.003605 |
| ACO1 | 24.01818 | 6.608743 | -1.86168 | 1.20E-41 | 1.06E-40 |
| ACSL3 | 26.82965 | 27.73455 | 0.047856 | 0.003142 | 0.003934 |
| ACSL4 | 13.55095 | 6.175778 | -1.1337 | 4.45E-42 | 4.13E-41 |
| ACVR1B | 9.62824 | 11.27817 | 0.228189 | 0.000888 | 0.001179 |
| ADAMTS13 | 0.693046 | 0.920665 | 0.409724 | 0.047143 | 0.053856 |
| AEBP2 | 6.215479 | 5.005241 | -0.31243 | 2.38E-11 | 4.58E-11 |
| AGPAT3 | 8.388966 | 10.61454 | 0.339477 | 1.17E-12 | 2.46E-12 |
| AGPS | 7.543905 | 7.167455 | -0.07385 | 0.000135 | 0.000187 |
| AHCY | 33.28266 | 56.54308 | 0.76458 | 1.25E-26 | 4.82E-26 |
| AKT1S1 | 7.325396 | 11.05899 | 0.594241 | 2.17E-31 | 1.06E-30 |
| ALDH3A2 | 21.74235 | 14.75263 | -0.55954 | 3.93E-24 | 1.35E-23 |
| ALOX5 | 6.641346 | 6.551011 | -0.01976 | 0.03536 | 0.040526 |
| AMN | 0.085797 | 0.107548 | 0.325979 | 0.000317 | 0.00043 |
| ANO6 | 33.88992 | 13.04018 | -1.37789 | 1.06E-48 | 1.62E-47 |
| AQP5 | 7.769035 | 9.447102 | 0.282136 | 2.29E-14 | 5.25E-14 |
| AR | 11.02037 | 15.72887 | 0.513242 | 0.002445 | 0.003126 |
| ARF6 | 37.92243 | 52.9474 | 0.481508 | 1.66E-19 | 4.88E-19 |
| ARNTL | 2.501116 | 2.175257 | -0.20139 | 0.003223 | 0.00402 |
| ARRDC3 | 51.51493 | 18.78694 | -1.45526 | 3.51E-40 | 2.69E-39 |
| ASAH2 | 0.115679 | 0.137548 | 0.249805 | 0.112138 | 0.123316 |
| ASNS | 4.297467 | 8.435726 | 0.973026 | 9.05E-16 | 2.17E-15 |
| ATF2 | 11.14755 | 10.05919 | -0.14821 | 1.83E-06 | 2.91E-06 |
| ATF4 | 121.4875 | 114.074 | -0.09084 | 3.65E-05 | 5.32E-05 |
| ATG13 | 14.33341 | 14.03385 | -0.03047 | 0.001321 | 0.00174 |
| ATG16L1 | 6.042773 | 8.830952 | 0.547358 | 3.75E-14 | 8.44E-14 |
| ATG3 | 6.986156 | 8.345838 | 0.256558 | 1.03E-12 | 2.18E-12 |
| ATG4D | 4.139888 | 7.632219 | 0.882511 | 6.31E-36 | 3.98E-35 |
| ATM | 3.525734 | 2.500691 | -0.4956 | 2.80E-16 | 6.90E-16 |
| ATP5MC3 | 18.83239 | 21.76506 | 0.208798 | 0.000209 | 0.000287 |
| AURKA | 1.117368 | 9.458792 | 3.081552 | 2.08E-62 | 2.44E-60 |
| BACH1 | 6.469392 | 5.497328 | -0.2349 | 3.22E-07 | 5.28E-07 |
| BAP1 | 14.9583 | 16.1339 | 0.109148 | 0.00624 | 0.007621 |
| BCAT2 | 5.406612 | 9.795035 | 0.857326 | 3.30E-21 | 1.02E-20 |
| BECN1 | 9.667438 | 10.65249 | 0.139985 | 0.231177 | 0.251094 |
| BID | 4.923679 | 6.151665 | 0.32124 | 7.73E-05 | 0.00011 |
| BRD2 | 36.172 | 40.05243 | 0.147016 | 0.006363 | 0.007745 |
| BRD3 | 6.483651 | 7.842693 | 0.274543 | 3.89E-05 | 5.65E-05 |
| BRD4 | 7.704801 | 9.765905 | 0.341996 | 3.41E-12 | 7.03E-12 |
| BRD7 | 7.826526 | 7.518005 | -0.05802 | 0.000111 | 0.000156 |
| BRPF1 | 5.508218 | 5.836223 | 0.083449 | 0.553977 | 0.575159 |
| CA9 | 0.155718 | 3.995788 | 4.68147 | 0.000522 | 0.000701 |
| CAMKK2 | 12.89647 | 11.62622 | -0.1496 | 1.50E-08 | 2.58E-08 |
| CAPG | 36.84575 | 77.02506 | 1.06383 | 8.61E-29 | 3.71E-28 |
| CAV1 | 187.7649 | 19.70001 | -3.25266 | 1.28E-65 | 4.53E-63 |
| CBR1 | 13.09496 | 13.95937 | 0.092223 | 0.252554 | 0.272635 |
| CBS | 0.034948 | 0.133349 | 1.931915 | 6.21E-07 | 1.02E-06 |
| CCDC6 | 17.72987 | 20.87437 | 0.235551 | 0.009133 | 0.011003 |
| CD44 | 41.26889 | 51.86676 | 0.329756 | 0.542975 | 0.565399 |
| CD82 | 18.37726 | 17.94831 | -0.03407 | 0.025776 | 0.02993 |
| CDC25A | 0.515268 | 2.208712 | 2.09981 | 1.31E-41 | 1.13E-40 |
| CDCA3 | 0.380158 | 2.976606 | 2.968999 | 3.25E-60 | 1.91E-58 |
| CDKN2A | 0.764672 | 5.127623 | 2.745377 | 1.76E-32 | 9.12E-32 |
| CEBPG | 12.62799 | 14.06414 | 0.155397 | 0.446765 | 0.469369 |
| CFL1 | 76.12614 | 152.2741 | 1.000207 | 4.75E-50 | 7.98E-49 |
| CGAS | 1.283353 | 2.416866 | 0.913219 | 3.44E-16 | 8.39E-16 |
| CHAC1 | 0.596787 | 1.578135 | 1.402932 | 1.33E-11 | 2.62E-11 |
| CHMP1A | 23.43627 | 32.65337 | 0.478489 | 1.84E-13 | 3.97E-13 |
| CHMP5 | 26.55882 | 35.90712 | 0.435079 | 5.87E-16 | 1.42E-15 |
| CHMP6 | 8.725007 | 10.7657 | 0.303213 | 9.18E-05 | 0.00013 |
| CHP1 | 39.14641 | 33.90221 | -0.2075 | 3.04E-06 | 4.71E-06 |
| CIRBP | 55.58915 | 45.0307 | -0.30389 | 2.80E-09 | 4.89E-09 |
| CISD1 | 5.292499 | 7.030727 | 0.409725 | 2.34E-14 | 5.32E-14 |
| CISD2 | 8.525504 | 13.03546 | 0.612585 | 1.38E-34 | 7.88E-34 |
| CISD3 | 7.35462 | 16.73505 | 1.18615 | 2.88E-27 | 1.14E-26 |
| COPZ1 | 38.55412 | 55.65032 | 0.529505 | 1.50E-27 | 6.09E-27 |
| COX4I2 | 4.661072 | 2.796769 | -0.7369 | 6.79E-15 | 1.59E-14 |
| CREB1 | 7.420461 | 6.146303 | -0.27179 | 4.11E-11 | 7.89E-11 |
| CREB3 | 24.60895 | 34.4452 | 0.48512 | 5.53E-17 | 1.40E-16 |
| CREB5 | 2.495738 | 0.62143 | -2.0058 | 3.36E-55 | 7.91E-54 |
| CS | 36.55245 | 28.90001 | -0.3389 | 6.29E-06 | 9.57E-06 |
| CTSB | 95.27191 | 136.7466 | 0.521382 | 7.31E-18 | 1.97E-17 |
| CYB5R1 | 16.26398 | 25.36617 | 0.641225 | 8.06E-19 | 2.26E-18 |
| CYBB | 13.92726 | 14.94756 | 0.101999 | 0.25209 | 0.272635 |
| CYGB | 12.05798 | 5.138866 | -1.23047 | 6.13E-34 | 3.33E-33 |
| DAZAP1 | 7.412091 | 11.09137 | 0.581485 | 1.62E-30 | 7.43E-30 |
| DCAF7 | 17.03986 | 25.1248 | 0.560199 | 2.92E-16 | 7.16E-16 |
| DDIT3 | 12.40788 | 13.80297 | 0.153722 | 0.391094 | 0.415831 |
| DDIT4 | 32.57816 | 43.11039 | 0.40413 | 0.000372 | 0.000501 |
| DDR2 | 28.09994 | 4.88543 | -2.52401 | 3.61E-53 | 7.50E-52 |
| DECR1 | 21.06929 | 20.03416 | -0.07268 | 0.014665 | 0.017313 |
| DHODH | 2.049193 | 1.635792 | -0.32507 | 1.13E-16 | 2.82E-16 |
| DLD | 19.33925 | 16.43228 | -0.235 | 2.05E-08 | 3.47E-08 |
| DNAJB6 | 7.201022 | 8.045527 | 0.159985 | 0.062068 | 0.07 |
| DPEP1 | 0.113216 | 0.619921 | 2.453001 | 2.07E-37 | 1.38E-36 |
| DPP4 | 6.326117 | 2.289468 | -1.46631 | 8.51E-36 | 5.27E-35 |
| DRD4 | 0.221603 | 0.598802 | 1.434101 | 2.11E-17 | 5.45E-17 |
| DUSP1 | 303.3424 | 80.13181 | -1.9205 | 1.29E-47 | 1.68E-46 |
| ECH1 | 52.64043 | 46.0406 | -0.19326 | 6.41E-06 | 9.72E-06 |
| EGLN2 | 2.959808 | 5.441259 | 0.878437 | 3.50E-13 | 7.49E-13 |
| EGR1 | 507.0948 | 82.87737 | -2.61321 | 2.39E-51 | 4.44E-50 |
| EIF2AK4 | 7.112492 | 6.686919 | -0.08901 | 9.41E-05 | 0.000133 |
| EIF2S1 | 14.09846 | 15.38496 | 0.125983 | 0.052931 | 0.060079 |
| ELAVL1 | 9.614376 | 14.71194 | 0.613723 | 2.76E-52 | 5.42E-51 |
| ELOVL5 | 104.2543 | 102.1688 | -0.02915 | 0.000251 | 0.000342 |
| EMC2 | 10.25239 | 12.64202 | 0.302266 | 0.004798 | 0.005902 |
| ENPP2 | 30.58315 | 4.924151 | -2.63479 | 5.86E-60 | 2.95E-58 |
| EPAS1 | 78.25282 | 21.68227 | -1.85163 | 1.11E-55 | 2.80E-54 |
| EZH2 | 1.340859 | 6.312203 | 2.234986 | 7.23E-58 | 2.13E-56 |
| FADS2 | 6.087616 | 18.03685 | 1.566998 | 2.16E-09 | 3.79E-09 |
| FANCD2 | 1.194586 | 3.145722 | 1.396881 | 3.49E-41 | 2.87E-40 |
| FAR1 | 9.741641 | 10.9412 | 0.167534 | 0.89869 | 0.90899 |
| FBXW7 | 4.207057 | 2.260469 | -0.89619 | 7.95E-48 | 1.12E-46 |
| FGF21 | 0.004792 | 0.069791 | 3.864298 | 0.7697 | 0.787548 |
| FH | 29.85847 | 46.27992 | 0.632246 | 2.50E-28 | 1.05E-27 |
| FOXO4 | 14.2929 | 7.311494 | -0.96706 | 1.11E-47 | 1.50E-46 |
| FTH1 | 268.6079 | 303.2277 | 0.1749 | 0.011223 | 0.013429 |
| FTL | 2596.077 | 2075.167 | -0.32311 | 0.014905 | 0.017538 |
| FXN | 3.154159 | 3.248516 | 0.042525 | 0.100367 | 0.110717 |
| FZD7 | 33.229 | 14.09407 | -1.23735 | 3.48E-30 | 1.58E-29 |
| G6PD | 14.45218 | 21.78686 | 0.592172 | 1.93E-19 | 5.64E-19 |
| GABARAPL1 | 29.04286 | 10.78095 | -1.4297 | 4.24E-62 | 3.01E-60 |
| GABARAPL2 | 29.22213 | 24.58752 | -0.24914 | 6.79E-18 | 1.84E-17 |
| GCLC | 6.738156 | 5.411647 | -0.31629 | 1.47E-13 | 3.20E-13 |
| GJA1 | 55.29418 | 101.9413 | 0.88254 | 0.029027 | 0.033595 |
| GLRX5 | 12.60449 | 17.00525 | 0.432043 | 5.59E-19 | 1.60E-18 |
| GLS2 | 0.147204 | 0.176581 | 0.262516 | 0.795551 | 0.809307 |
| GOT1 | 14.05447 | 19.4636 | 0.46975 | 1.24E-25 | 4.57E-25 |
| GPT2 | 11.22971 | 7.672597 | -0.54953 | 1.43E-11 | 2.81E-11 |
| GPX4 | 151.0102 | 126.8235 | -0.25182 | 0.011999 | 0.014309 |
| GSK3B | 9.677026 | 11.97384 | 0.307251 | 1.61E-08 | 2.76E-08 |
| GSTZ1 | 2.501535 | 3.093853 | 0.306591 | 0.016623 | 0.019495 |
| HCAR1 | 12.92403 | 7.993866 | -0.69309 | 9.72E-18 | 2.58E-17 |
| HDDC3 | 3.17937 | 3.816318 | 0.263441 | 0.002213 | 0.002851 |
| HELLS | 0.618304 | 1.957677 | 1.662753 | 1.03E-41 | 9.33E-41 |
| HIC1 | 2.164626 | 1.362343 | -0.66803 | 2.90E-20 | 8.76E-20 |
| HIF1A | 21.36175 | 26.71767 | 0.322764 | 0.26759 | 0.28711 |
| HILPDA | 11.20925 | 17.92475 | 0.677263 | 5.46E-11 | 1.04E-10 |
| HRAS | 10.21697 | 17.49133 | 0.775672 | 1.44E-15 | 3.42E-15 |
| HSD17B11 | 21.52742 | 8.993463 | -1.25923 | 7.39E-45 | 8.16E-44 |
| HSF1 | 12.39342 | 20.46809 | 0.723803 | 4.16E-22 | 1.35E-21 |
| HSPA5 | 123.3076 | 210.1172 | 0.768933 | 1.34E-36 | 8.75E-36 |
| HSPB1 | 117.6245 | 352.5494 | 1.583636 | 2.47E-24 | 8.64E-24 |
| IDH1 | 32.16817 | 25.28384 | -0.34742 | 5.78E-14 | 1.29E-13 |
| IDH2 | 34.93088 | 85.28995 | 1.287873 | 2.60E-38 | 1.83E-37 |
| IL33 | 25.32936 | 3.979691 | -2.67008 | 4.26E-62 | 3.01E-60 |
| INTS2 | 2.755524 | 3.342433 | 0.278572 | 0.781766 | 0.797583 |
| IREB2 | 7.313791 | 6.099187 | -0.262 | 9.81E-11 | 1.82E-10 |
| ISCU | 22.03966 | 15.91442 | -0.46977 | 2.83E-33 | 1.51E-32 |
| JDP2 | 5.707048 | 4.036855 | -0.49951 | 2.45E-12 | 5.09E-12 |
| JUN | 163.6964 | 62.92235 | -1.37938 | 2.78E-38 | 1.93E-37 |
| KDM3B | 18.15012 | 15.72829 | -0.20662 | 1.98E-06 | 3.13E-06 |
| KDM4A | 11.50113 | 13.67928 | 0.250216 | 4.05E-06 | 6.22E-06 |
| KDM5A | 7.991032 | 7.556138 | -0.08073 | 0.001445 | 0.00189 |
| KDM5C | 13.8819 | 18.0833 | 0.381453 | 5.80E-14 | 1.29E-13 |
| KDM6B | 8.199466 | 7.037462 | -0.22047 | 2.53E-06 | 3.93E-06 |
| KEAP1 | 16.06499 | 21.94486 | 0.449963 | 1.06E-17 | 2.80E-17 |
| KIF20A | 0.819235 | 8.072555 | 3.300676 | 4.23E-59 | 1.66E-57 |
| KLF2 | 42.3952 | 15.88498 | -1.41624 | 2.82E-34 | 1.56E-33 |
| KLHDC3 | 49.65239 | 56.04232 | 0.174653 | 0.947571 | 0.955694 |
| KLHL24 | 9.851377 | 7.39557 | -0.41366 | 1.33E-18 | 3.69E-18 |
| KMT2D | 6.513876 | 6.008497 | -0.11651 | 0.009436 | 0.011329 |
| KRAS | 5.850845 | 9.255447 | 0.661658 | 5.57E-23 | 1.86E-22 |
| LAMP2 | 42.67848 | 48.98126 | 0.198721 | 0.050905 | 0.057967 |
| LGMN | 45.23462 | 32.53147 | -0.47559 | 1.41E-05 | 2.09E-05 |
| LIFR | 18.00372 | 3.170446 | -2.50554 | 1.29E-62 | 2.27E-60 |
| LIG3 | 2.334103 | 4.204432 | 0.849043 | 3.99E-27 | 1.57E-26 |
| LONP1 | 11.84477 | 18.54709 | 0.646942 | 1.17E-21 | 3.67E-21 |
| LPCAT3 | 4.764935 | 4.996748 | 0.068533 | 0.867771 | 0.880239 |
| LPIN1 | 6.479474 | 3.367039 | -0.9444 | 1.60E-35 | 9.42E-35 |
| LRRFIP1 | 13.03573 | 15.09954 | 0.212034 | 2.22E-05 | 3.25E-05 |
| LYRM1 | 10.6213 | 8.073688 | -0.39566 | 1.51E-17 | 3.95E-17 |
| MAFG | 6.715674 | 5.626541 | -0.25528 | 8.51E-14 | 1.87E-13 |
| MAP1LC3A | 8.940806 | 11.0922 | 0.311069 | 0.152196 | 0.166332 |
| MAP3K11 | 6.533663 | 7.706645 | 0.238211 | 0.000108 | 0.000152 |
| MAP3K14 | 4.496242 | 3.376852 | -0.41304 | 1.20E-10 | 2.22E-10 |
| MAP3K5 | 8.669245 | 4.602977 | -0.91334 | 1.52E-35 | 9.12E-35 |
| MAPK1 | 14.78094 | 13.28802 | -0.15361 | 1.01E-06 | 1.63E-06 |
| MAPK14 | 11.84032 | 10.26498 | -0.20598 | 7.02E-13 | 1.49E-12 |
| MAPK3 | 25.16101 | 25.06425 | -0.00556 | 0.03137 | 0.036189 |
| MAPK8 | 4.757418 | 4.236342 | -0.16736 | 0.000124 | 0.000174 |
| MAPK9 | 5.754007 | 7.683736 | 0.417241 | 2.97E-14 | 6.71E-14 |
| MDM2 | 6.414413 | 8.505815 | 0.407132 | 0.005474 | 0.006709 |
| MDM4 | 4.559036 | 5.740702 | 0.332498 | 0.002194 | 0.002837 |
| MEF2C | 6.250185 | 2.792961 | -1.1621 | 3.09E-45 | 3.52E-44 |
| METTL14 | 5.49215 | 4.57853 | -0.26249 | 6.34E-11 | 1.20E-10 |
| MFN2 | 29.54161 | 25.04349 | -0.23831 | 4.26E-12 | 8.69E-12 |
| MIB1 | 9.699765 | 9.673084 | -0.00397 | 0.144962 | 0.158917 |
| MIB2 | 2.265421 | 2.451767 | 0.114042 | 0.615084 | 0.634868 |
| MLLT1 | 15.51555 | 19.05079 | 0.296135 | 1.62E-10 | 2.96E-10 |
| MLST8 | 6.856441 | 12.24885 | 0.837114 | 5.46E-36 | 3.50E-35 |
| MPC1 | 21.88551 | 16.83246 | -0.37873 | 6.25E-18 | 1.71E-17 |
| MTCH1 | 55.85462 | 61.25253 | 0.133093 | 0.627137 | 0.645421 |
| MTDH | 22.57961 | 33.63117 | 0.574779 | 3.95E-12 | 8.11E-12 |
| MTF1 | 4.611788 | 3.861928 | -0.256 | 7.62E-10 | 1.37E-09 |
| MTOR | 7.406889 | 6.97643 | -0.08638 | 0.003043 | 0.003822 |
| MUC1 | 24.10442 | 130.0018 | 2.431162 | 1.62E-30 | 7.43E-30 |
| MYB | 9.172861 | 17.75998 | 0.953187 | 9.04E-12 | 1.80E-11 |
| NCF2 | 4.944009 | 6.160227 | 0.317302 | 0.000663 | 0.000886 |
| NCOA3 | 8.875055 | 10.72311 | 0.272896 | 0.074497 | 0.08375 |
| NCOA4 | 77.26619 | 58.84908 | -0.39282 | 3.04E-24 | 1.05E-23 |
| NDRG1 | 29.81754 | 31.68195 | 0.0875 | 9.94E-12 | 1.97E-11 |
| NEDD4 | 3.600615 | 2.79729 | -0.36421 | 2.32E-11 | 4.50E-11 |
| NEDD4L | 6.839111 | 5.522885 | -0.30839 | 9.50E-10 | 1.69E-09 |
| NF2 | 6.233444 | 6.598443 | 0.082096 | 0.960276 | 0.963004 |
| NFE2L2 | 26.8584 | 19.24545 | -0.48086 | 1.40E-31 | 7.08E-31 |
| NFS1 | 3.795011 | 5.290609 | 0.47933 | 2.16E-18 | 5.95E-18 |
| NGB | 0.012107 | 0.104109 | 3.104213 | 7.69E-15 | 1.79E-14 |
| NNMT | 46.24453 | 32.08315 | -0.52747 | 2.20E-12 | 4.60E-12 |
| NOX1 | 0.263673 | 0.514602 | 0.964708 | 9.26E-25 | 3.33E-24 |
| NOX4 | 1.49585 | 1.824961 | 0.2869 | 0.000146 | 0.000201 |
| NR1D2 | 17.9675 | 9.954683 | -0.85194 | 1.33E-26 | 5.06E-26 |
| NR5A2 | 1.478529 | 0.594219 | -1.3151 | 2.37E-45 | 2.79E-44 |
| NRAS | 13.30857 | 17.97031 | 0.43326 | 1.07E-06 | 1.72E-06 |
| NT5DC2 | 7.791823 | 11.89716 | 0.610584 | 7.25E-05 | 0.000104 |
| NUPR1 | 18.95458 | 25.98723 | 0.455257 | 0.008406 | 0.010162 |
| OSBPL9 | 13.42066 | 9.237032 | -0.53895 | 1.08E-27 | 4.42E-27 |
| OTUB1 | 11.29733 | 18.16511 | 0.685188 | 1.68E-34 | 9.41E-34 |
| OXSR1 | 10.91818 | 10.15619 | -0.10437 | 1.73E-05 | 2.54E-05 |
| P4HB | 100.6255 | 194.994 | 0.954433 | 2.22E-40 | 1.78E-39 |
| PANX1 | 6.797962 | 9.069309 | 0.41589 | 9.64E-11 | 1.80E-10 |
| PANX2 | 0.614938 | 1.995162 | 1.697992 | 6.24E-14 | 1.38E-13 |
| PAQR3 | 1.410689 | 1.116057 | -0.33799 | 1.50E-11 | 2.92E-11 |
| PARK7 | 65.87739 | 83.77128 | 0.346672 | 5.02E-12 | 1.02E-11 |
| PARP1 | 18.67829 | 43.0327 | 1.204071 | 7.12E-56 | 1.93E-54 |
| PARP10 | 7.194602 | 15.94512 | 1.148128 | 1.12E-20 | 3.44E-20 |
| PARP11 | 2.872026 | 2.600058 | -0.14352 | 4.46E-05 | 6.45E-05 |
| PARP14 | 8.109136 | 14.03075 | 0.790972 | 5.40E-12 | 1.09E-11 |
| PARP15 | 0.486724 | 0.581336 | 0.256269 | 0.055914 | 0.063262 |
| PARP2 | 5.32623 | 6.14245 | 0.2057 | 0.00228 | 0.002927 |
| PARP3 | 10.89416 | 8.325119 | -0.38801 | 3.31E-15 | 7.80E-15 |
| PARP4 | 15.34674 | 12.9015 | -0.25039 | 2.88E-10 | 5.21E-10 |
| PARP9 | 6.881679 | 13.82885 | 1.006849 | 1.45E-28 | 6.16E-28 |
| PCK2 | 10.0172 | 15.16032 | 0.597821 | 1.59E-17 | 4.14E-17 |
| PDK4 | 120.4869 | 11.59866 | -3.37685 | 3.64E-58 | 1.17E-56 |
| PDSS2 | 5.518248 | 5.778852 | 0.066573 | 0.002096 | 0.00272 |
| PEBP1 | 194.4992 | 205.326 | 0.078152 | 0.540751 | 0.564749 |
| PEX12 | 4.691805 | 4.456672 | -0.07418 | 0.001344 | 0.001764 |
| PEX2 | 10.36547 | 12.35015 | 0.252743 | 0.004652 | 0.005742 |
| PEX3 | 9.181599 | 7.631242 | -0.26683 | 1.44E-10 | 2.65E-10 |
| PEX6 | 11.8133 | 13.78575 | 0.222765 | 0.092628 | 0.102501 |
| PGD | 48.6043 | 48.76866 | 0.00487 | 0.711445 | 0.730058 |
| PGRMC1 | 85.53262 | 89.03195 | 0.057848 | 0.280726 | 0.300292 |
| PHF21A | 4.61313 | 4.184076 | -0.14084 | 6.62E-07 | 1.08E-06 |
| PHKG2 | 2.429659 | 5.284014 | 1.12088 | 9.42E-50 | 1.51E-48 |
| PIEZO1 | 12.87458 | 12.26993 | -0.0694 | 0.002901 | 0.00367 |
| PIR | 5.819432 | 5.458944 | -0.09226 | 1.29E-05 | 1.94E-05 |
| PLA2G6 | 2.607596 | 2.203862 | -0.24269 | 1.03E-08 | 1.79E-08 |
| PLIN2 | 23.25541 | 12.59566 | -0.88464 | 1.09E-24 | 3.83E-24 |
| PML | 7.201956 | 6.61387 | -0.12289 | 2.47E-08 | 4.17E-08 |
| POR | 10.37723 | 15.22912 | 0.55341 | 3.05E-13 | 6.56E-13 |
| PPARA | 3.252204 | 1.317925 | -1.30315 | 1.37E-46 | 1.73E-45 |
| PPARD | 9.235819 | 11.68047 | 0.338787 | 3.49E-06 | 5.37E-06 |
| PPP1R13L | 9.452211 | 10.18577 | 0.107831 | 0.982141 | 0.982141 |
| PRDX1 | 128.2367 | 250.7738 | 0.967577 | 1.59E-39 | 1.17E-38 |
| PRDX6 | 136.4459 | 103.4828 | -0.39894 | 5.35E-06 | 8.17E-06 |
| PRKAA1 | 14.74748 | 9.24745 | -0.67334 | 2.61E-28 | 1.08E-27 |
| PRKAA2 | 1.766643 | 2.007227 | 0.184193 | 0.258048 | 0.277717 |
| PRKCA | 2.017684 | 0.95955 | -1.07227 | 4.16E-43 | 4.08E-42 |
| PROM2 | 13.18844 | 23.4192 | 0.828418 | 1.14E-15 | 2.72E-15 |
| PRR5 | 3.315513 | 2.729822 | -0.28043 | 1.11E-06 | 1.77E-06 |
| PSAT1 | 4.75373 | 8.096088 | 0.768165 | 5.43E-12 | 1.09E-11 |
| PTEN | 14.7555 | 9.556773 | -0.62666 | 4.85E-33 | 2.56E-32 |
| PTPN18 | 14.56508 | 16.77104 | 0.203458 | 0.001132 | 0.001497 |
| PTPN6 | 5.987473 | 11.28523 | 0.914416 | 8.89E-35 | 5.14E-34 |
| RARRES2 | 59.86038 | 23.4817 | -1.35006 | 9.53E-25 | 3.40E-24 |
| RB1 | 11.27385 | 9.829162 | -0.19784 | 1.35E-05 | 2.01E-05 |
| RBMS1 | 9.621554 | 5.219475 | -0.88237 | 3.30E-41 | 2.77E-40 |
| RELA | 18.06153 | 19.34075 | 0.098724 | 0.032693 | 0.037591 |
| RICTOR | 4.357782 | 3.397966 | -0.35892 | 1.11E-09 | 1.97E-09 |
| RIPK1 | 13.39719 | 12.62004 | -0.08621 | 0.000667 | 0.000888 |
| RPL8 | 548.3132 | 753.4934 | 0.458595 | 2.33E-06 | 3.64E-06 |
| RPTOR | 4.341539 | 5.071186 | 0.224117 | 0.006455 | 0.00783 |
| RRM2 | 1.194881 | 11.98794 | 3.326646 | 9.89E-59 | 3.49E-57 |
| SAT1 | 77.97509 | 100.3713 | 0.364262 | 0.014563 | 0.017251 |
| SELENOS | 11.87335 | 18.11983 | 0.609843 | 5.88E-31 | 2.80E-30 |
| SENP1 | 3.975847 | 4.800711 | 0.271986 | 1.47E-05 | 2.18E-05 |
| SETD1B | 5.922719 | 6.63411 | 0.163643 | 0.003718 | 0.004622 |
| SIAH2 | 14.16969 | 44.58376 | 1.65371 | 1.20E-38 | 8.63E-38 |
| SIRT1 | 10.47378 | 7.886375 | -0.40935 | 2.51E-19 | 7.27E-19 |
| SIRT2 | 10.71515 | 8.632377 | -0.31182 | 5.80E-22 | 1.86E-21 |
| SIRT3 | 6.247778 | 5.500403 | -0.18381 | 8.02E-06 | 1.21E-05 |
| SIRT6 | 3.213076 | 5.949189 | 0.888738 | 1.51E-31 | 7.50E-31 |
| SLC11A2 | 8.497043 | 8.928187 | 0.071406 | 0.953588 | 0.959022 |
| SLC16A1 | 6.604228 | 8.108187 | 0.295989 | 6.03E-05 | 8.65E-05 |
| SLC1A4 | 8.702111 | 17.07883 | 0.972772 | 7.58E-18 | 2.03E-17 |
| SLC1A5 | 43.01034 | 48.62849 | 0.177118 | 0.445596 | 0.469369 |
| SLC25A28 | 18.28741 | 17.13 | -0.09433 | 2.02E-06 | 3.18E-06 |
| SLC2A1 | 9.522344 | 25.89177 | 1.443105 | 1.26E-31 | 6.46E-31 |
| SLC2A12 | 1.363592 | 0.614639 | -1.1496 | 7.34E-31 | 3.45E-30 |
| SLC2A3 | 9.342973 | 5.382659 | -0.79556 | 3.51E-08 | 5.90E-08 |
| SLC2A6 | 0.674501 | 2.060263 | 1.610935 | 6.81E-26 | 2.56E-25 |
| SLC2A8 | 3.996774 | 5.034065 | 0.332888 | 1.49E-05 | 2.20E-05 |
| SLC38A1 | 27.20271 | 41.24471 | 0.600459 | 9.77E-07 | 1.58E-06 |
| SLC39A14 | 11.35244 | 10.10733 | -0.1676 | 1.96E-07 | 3.24E-07 |
| SLC39A7 | 41.21569 | 79.51924 | 0.94811 | 3.48E-40 | 2.69E-39 |
| SLC3A2 | 25.52308 | 38.6603 | 0.59905 | 1.17E-19 | 3.46E-19 |
| SLC40A1 | 63.83961 | 84.42642 | 0.403243 | 0.002946 | 0.003714 |
| SLC7A11 | 0.261791 | 1.14646 | 2.130702 | 2.00E-22 | 6.54E-22 |
| SLC7A5 | 5.08341 | 28.73826 | 2.499105 | 8.23E-30 | 3.68E-29 |
| SMAD7 | 10.04248 | 9.763544 | -0.04064 | 0.091011 | 0.101028 |
| SMG9 | 3.327985 | 4.939824 | 0.569811 | 1.89E-22 | 6.24E-22 |
| SMPD1 | 13.01637 | 14.40328 | 0.14607 | 0.086039 | 0.09581 |
| SNCA | 2.122953 | 0.614472 | -1.78865 | 2.47E-53 | 5.45E-52 |
| SNX4 | 15.28573 | 17.70942 | 0.212332 | 2.32E-06 | 3.64E-06 |
| SNX5 | 14.41029 | 12.95165 | -0.15396 | 1.38E-09 | 2.43E-09 |
| SOCS1 | 1.912059 | 3.117035 | 0.705047 | 0.013035 | 0.015493 |
| SP1 | 23.70498 | 20.19548 | -0.23116 | 2.98E-09 | 5.18E-09 |
| SRC | 6.032619 | 10.6722 | 0.823001 | 1.38E-23 | 4.68E-23 |
| SREBF1 | 19.14768 | 43.19774 | 1.173786 | 7.19E-19 | 2.03E-18 |
| SREBF2 | 19.26695 | 22.77328 | 0.241214 | 0.024043 | 0.028011 |
| SRSF9 | 13.77907 | 22.02413 | 0.676607 | 8.67E-38 | 5.88E-37 |
| SRXN1 | 0.342881 | 0.682511 | 0.993143 | 2.83E-27 | 1.14E-26 |
| STAT3 | 38.684 | 38.23139 | -0.01698 | 0.077269 | 0.08659 |
| STEAP3 | 14.11621 | 13.18866 | -0.09805 | 0.000325 | 0.00044 |
| STK11 | 4.862469 | 6.672928 | 0.456631 | 8.87E-11 | 1.67E-10 |
| STMN1 | 10.74601 | 36.0034 | 1.744332 | 2.56E-46 | 3.12E-45 |
| SUV39H1 | 2.612356 | 4.361486 | 0.739468 | 3.92E-31 | 1.90E-30 |
| TBK1 | 7.075317 | 8.489564 | 0.262896 | 1.44E-07 | 2.39E-07 |
| TFAM | 9.966658 | 10.04692 | 0.011571 | 0.446097 | 0.469369 |
| TFAP2A | 9.454644 | 16.75387 | 0.8254 | 9.77E-15 | 2.25E-14 |
| TFAP2C | 34.30109 | 22.22854 | -0.62584 | 5.15E-08 | 8.58E-08 |
| TFRC | 10.2249 | 21.85738 | 1.096034 | 2.97E-17 | 7.59E-17 |
| TGFB1 | 9.826729 | 17.71795 | 0.850428 | 4.77E-23 | 1.60E-22 |
| TGFBR1 | 12.57913 | 12.74216 | 0.018577 | 0.003809 | 0.004718 |
| TIMM9 | 18.21099 | 17.45632 | -0.06106 | 0.083712 | 0.093514 |
| TIMP1 | 101.8058 | 218.7855 | 1.103698 | 7.02E-25 | 2.56E-24 |
| TLR4 | 9.502818 | 3.610748 | -1.39606 | 3.44E-42 | 3.28E-41 |
| TMBIM4 | 13.50119 | 15.88393 | 0.234481 | 0.002463 | 0.003138 |
| TMSB4X | 581.9121 | 638.4185 | 0.133701 | 0.303535 | 0.323709 |
| TNFAIP3 | 9.043743 | 8.293664 | -0.12491 | 0.000142 | 0.000197 |
| TOR2A | 2.250475 | 4.965742 | 1.14178 | 5.14E-44 | 5.33E-43 |
| TRIB3 | 4.884897 | 14.72333 | 1.591704 | 4.41E-44 | 4.72E-43 |
| TRIM46 | 0.520004 | 1.440061 | 1.469534 | 5.36E-20 | 1.60E-19 |
| TSC1 | 5.709381 | 4.256361 | -0.42371 | 2.13E-20 | 6.47E-20 |
| TSC22D3 | 54.41573 | 37.3915 | -0.54131 | 3.76E-10 | 6.77E-10 |
| TUBE1 | 2.533104 | 1.645391 | -0.62248 | 8.77E-30 | 3.87E-29 |
| TXN | 83.24766 | 157.998 | 0.924425 | 9.07E-36 | 5.52E-35 |
| TXNIP | 624.6027 | 163.5571 | -1.93314 | 3.32E-59 | 1.46E-57 |
| TXNRD1 | 11.79182 | 14.6745 | 0.315525 | 0.022479 | 0.026275 |
| TYRO3 | 4.349452 | 1.091595 | -1.9944 | 3.13E-29 | 1.36E-28 |
| UBC | 192.0985 | 181.4338 | -0.0824 | 0.001504 | 0.001959 |
| ULK1 | 7.500074 | 9.935023 | 0.405619 | 8.69E-11 | 1.64E-10 |
| ULK2 | 4.327671 | 4.02376 | -0.10505 | 3.63E-08 | 6.08E-08 |
| USP7 | 18.79487 | 24.89024 | 0.405241 | 6.18E-17 | 1.56E-16 |
| VCP | 56.70813 | 77.19234 | 0.444902 | 6.45E-27 | 2.50E-26 |
| VDAC2 | 21.87193 | 23.41 | 0.098044 | 0.577832 | 0.598167 |
| VDR | 7.201334 | 9.107599 | 0.338807 | 5.05E-05 | 7.27E-05 |
| VEGFA | 5.315826 | 9.166845 | 0.786132 | 2.60E-10 | 4.73E-10 |
| VLDLR | 7.40865 | 2.398705 | -1.62695 | 9.38E-40 | 7.04E-39 |
| WIPI1 | 9.375625 | 12.40287 | 0.403687 | 0.000196 | 0.000269 |
| WIPI2 | 9.767768 | 10.01868 | 0.036592 | 0.40816 | 0.432674 |
| WWTR1 | 15.59169 | 10.27333 | -0.60187 | 7.64E-26 | 2.84E-25 |
| YAP1 | 36.66073 | 17.40907 | -1.0744 | 1.72E-50 | 3.03E-49 |
| YTHDC2 | 4.24979 | 4.536147 | 0.094076 | 0.535395 | 0.560814 |
| YWHAE | 129.6324 | 140.9711 | 0.120973 | 0.175281 | 0.19097 |
| YY1AP1 | 12.48112 | 17.50428 | 0.48796 | 9.91E-22 | 3.15E-21 |
| ZEB1 | 8.116263 | 4.356354 | -0.89769 | 3.15E-21 | 9.83E-21 |
| ZFP36 | 342.0425 | 79.30431 | -2.1087 | 1.56E-43 | 1.57E-42 |
| ZFP69B | 0.72708 | 1.338087 | 0.879986 | 6.66E-19 | 1.89E-18 |
